# Medical Students' Syndrome among Medical Students in the University of Sharjah, UAE

**DOI:** 10.1055/s-0043-1768683

**Published:** 2023-06-07

**Authors:** Saud Hilmi Abdullah, Ahmed Ehab Ahmed, Homam Galal Eldin Algebail, Ahmed Fatooh, Laila Nour Aldeen Ismail, Nada Khaled Radwan, Nour Bade Sakan, Hiba Jawdat Barqawi

**Affiliations:** 1 College of Medicine, University of Sharjah, Sharjah, United Arab Emirates; 2 Department of Clinical Sciences, College of Medicine, University of Sharjah, Sharjah, United Arab Emirates

**Keywords:** health anxiety, knowledge, medical students' syndrome

## Abstract

**Background** Medical students' syndrome (MSS) is a set of psychosomatic symptoms that affect students due to their medical background knowledge.

**Objectives** This study aims to measure the prevalence and assess the knowledge about MSS among medical students at the University of Sharjah. It also aims to compare the attributed characteristics of MSS between different years of study.

**Methods** A self-administered 24-item questionnaire was distributed to 503 students enrolled in the College of Medicine at University of Sharjah using nonprobability convenience sampling during June 2021. A total of 472 responses were completed and analyzed using descriptive studies and chi-squared test.

**Results** The prevalence of MSS in this study was found to be 70.8% ( *n* = 334). Clinical year students were 1.75 times more likely to have felt they had a disease they studied about (95% confidence interval: 1.05–2.90, *p* = 0.03).

**Conclusion** Medical students are more likely to experience MSS as their education progresses.

## Introduction

Medical students' syndrome (MSS) is a combination of psychosomatic symptoms that occur due to students correlating their vague symptoms to medical conditions they recently learned about, 1 whether in their books, lectures, or hospital wards. 2 A study conducted in London has revealed that this phenomenon mainly occurs due to the extensive requirements the average medical student must meet during their preclinical and clinical years. These include long working hours, assignments, constant exam preparations, and the competitive environment they are immersed in. 2 3

The condition develops when a student hears about or studies a certain disease. The student then starts to assume they are experiencing symptoms related to the disease, without the presence of any underlying pathology. 4 Symptoms experienced might vary as students tend to change the disease they assume to have depending on their current clinical rotation. 4

MSS can be commonly confused with a condition known as health anxiety, which involves extensive worry about ones' health. 5 Health anxiety is different from MSS in terms of the time at which the person believes they are suffering from the disease. Health anxiety usually involves the fear of acquiring the disease in the future, whereas MSS involves the fear of already having the disease. 4

Multiple recent studies on MSS have focused on the help-seeking behavior of medical students in response to the symptoms they experience. 2 A study conducted in Karachi compared the help-seeking behavior between two groups, medical students and engineering students, and concluded that there is no significant difference between the two groups. 1 This is contrary to the popular belief that medical students tend to avoid seeking medical help when necessary.

MSS is a prevalent phenomenon among medical students. A study conducted in London reported a prevalence of up to 70% among medical students involved. 2 On the other hand, there is a scarcity of data regarding the prevalence of MSS in the United Arab Emirates (UAE). This study aims to measure the prevalence and assess the knowledge of MSS among medical students in the University of Sharjah. It also aims to compare and contrast attributed characteristics of MSS between different years of study.

## Methods

### Study Design

This cross-sectional study was conducted at the University of Sharjah, UAE. A self-administered questionnaire was developed after a thorough literature review and distributed using Microsoft Forms, where a link was sent to students via social media platforms. Non-English speakers were not considered as suitable candidates since the questionnaire was designed in English. Additionally, students in other majors such as engineering, dentistry, and health sciences were excluded from the study. Ethics approval was obtained from the Research Ethics Committee of the University of Sharjah (REC-21–06–01–02-S).

### Sample Size

A nonprobability convenience sampling approach was used to recruit participants from the College of Medicine, including foundation, preclinical (year 1–3), and clinical students (year 4 and 5). The sample size was calculated using the following formula:  , where *n* is the sample size, *p* is the expected prevalence, and SE is the sampling error. A margin of error of 5%, along with a confidence level of 95%, was predetermined for the calculation. The expected prevalence was approximated to be 10%, based on a previous study done in the UAE. 8 The sample size was then adjusted using the formula:  , where new ss is the new sample size, ss is the sample size, and pop is the population size since the total population is under 20,000. Accordingly, a minimum of 118 students were required for the study. Since there is a lack of a validated scale for measuring the prevalence of MSS, medical students who felt they had a disease they studied about were accounted for in the prevalence of this study, as per the definition of the syndrome.

### Questionnaire Development

Due to the absence of a specific tool for MSS, a questionnaire was developed after a thorough review of literature and other studies conducted around the world. The questionnaire consisted of 17 questions with a total of 24 items comprising of three main sections: demographics (6), prevalence and knowledge of MSS (3), and attributes of MSS (8). The first section collected demographic data regarding age, gender, nationality, year of study, and the presence of long-standing illnesses. Multiple-choice questions were used to investigate whether the participants had heard about MSS, if they felt they had a disease they studied about, and the duration of the feeling. In addition, a 4-point Likert scale ranging from never to always was employed to assess the attributes of MSS among students. These evaluated the effect of the syndrome on daily activities, sleep, intake of medications, noticing bodily changes, looking up symptoms online, and talking to people about these symptoms. No identification questions were asked to ensure anonymity of the participants.

### Data Collection

A pilot study was conducted on 30 participants prior to data collection. Based on the feedback received, the questionnaire was modified and tested again. Nonprobability convenience sampling was utilized by generating a link to the questionnaire and sending it to students through social media platforms and email. Data collection took place over a 3-week timeframe during June 2021. An information sheet was presented prior to starting the questionnaire. Answering the questions indicated the students' consent to participate in the study. Data collected were only accessible by the investigators to maintain confidentiality.

### Statistical Analysis

Data analysis was conducted using IBM SPSS statistical software version 25. Descriptive statistics and chi-squared tests were used to analyze the data. The normality of data distribution was tested using the Kolmogorov–Smirnov test and Q-Q plots. Frequencies and odds ratios were reported as applicable, and valid percentages were calculated to account for missing data. Additionally, means and standard deviations were reported for quantitative variables. A scoring system called the MSS score was generated using multiple questions from the prevalence and attributes of MSS sections, resulting in a range of 0 to 23. Correct answers were given a point, while incorrect or skipped questions received no points. The scores were then grouped into four categories: none (0), mild (1–8), moderate (9–16), and severe (17–23). Furthermore, 4-point Likert scales were collapsed into two-point scales and were then used accordingly in the analysis. A *p* -value of less than 0.05 was considered significant.

## Results

### Demographics

A total of 472 out of 503 questionnaires were complete and analyzed in this study. The mean age of the participants was 20.6 years, with a minimum age of 17 and a maximum of 25 years. Females comprised the majority of the sample at 71% ( *n* = 335), while the nationalities of the participants included Arabs, Emiratis, and non-Arabs. The sample consisted of foundation year students, preclinical students, and clinical students ( Table 1 provides further details).

**Table 1:** Demographic distribution of study participants

Demographics		Count	Percentage
Age	17	7	1.5
	18	47	9.9
	19	97	20.6
	20	89	18.8
	21	82	17.4
	22	74	15.7
	23	60	12.7
	24	15	3.2
	25 +	1	0.2
Gender	Male	137	29.0
	Female	335	71.0
Nationality	UAE national	103	21.8
	Arab	343	72.7
	Non-Arab	26	5.5
Year	Foundation medicine	67	14.2
	Year 1	83	17.6
	Year 2	91	19.3
	Year 3	97	20.6
	Year 4	88	18.7
	Year 5	45	9.6

Upon analyzing the data, it was found that clinical year students were 2.49 times more likely than preclinical year students to consider their field of study as a reason of acquiring an illness (95% confidence interval [CI]: 1.23–5.05, *p* = 0.01), *X* ^2^ (1, *N* = 162) = 6.6, *p* = 0.01.

### Prevalence and Knowledge

The prevalence of MSS in the study population is 70.8% ( *n* = 334). Among the students surveyed, 55.9% had previously heard about MSS ( *n* = 264). Students in the age range of 21 to 25 were 2.02 times more likely to have felt they had a disease they studied about than students aged between 16 and 20 (95% CI: 1.34–3.03, *p* = 0.001), *X* ^2^ (1, *N* = 472) = 11.6, *p* = 0.001. Females were 1.86 times more likely to have felt that they had a disease they studied about when compared with males (95% CI: 1.22–2.83, *p* = 0.004), *X* ^2^ (1, *N* = 472) = 8.3, *p* = 0.004. Clinical students were 1.75 times more likely to have felt they had a disease they studied about when compared with preclinical students (95% CI: 1.05–2.90, *p* = 0.03). *X* ^2^ (1, *N* = 404) = 4.7, *p* = 0.03, and 4.72 times more likely when compared with foundation year students (95% CI: 2.48–9.01, *p* < 0.0005), *X* ^2^ (1, *N* = 200) = 23.7, *p* < 0.001. Students who were aware of MSS were 2.93 times more likely to experience symptoms than those who were not (95% CI: 1.94–4.42, *p* < 0.0005), *X* ^2^ (1, *N* = 471) = 27.1, *p* < 0.001.

### Attributes

Participants who felt they had a disease they studied about were asked further questions to elaborate on their feelings. It was found that 1.5% of them took self-prescribed medications ( *n* = 5), 24.3% were able to subdue their thoughts ( *n* = 81), and 6.3% were bothered by the feeling ( *n* = 21). Out of all participants, 28% sought medical assistance ( *n* = 92), and 37% mentioned that they were diagnosed with presumed disease ( *n* = 34). Students who thought that the feeling hindered their daily activities or disturbed their sleep were 5.88 times (95% CI: 2.82–12.20, *p* < 0.0005), *X* ^2^ (1, *N* = 334) = 26.7, *p* = 0.001, and 2.77 times (95% CI: 1.49–5.15, *p* = 0.001), *X* ^2^ (1, *N* = 334) = 10.8, *p* < 0.001, more likely to seek medical assistance, respectively.

Preclinical students were 1.80 times more likely to look up their symptoms online in comparison to clinical students (95% CI: 1.18–2.74, *p* = 0.006), *X* ^2^ (1, *N* = 404) = 7.5, *p* = 0.006. Furthermore, students who looked up their symptoms online were 2.33 times more likely to think they had an undiscovered illness (95% CI: 1.56–3.47, *p* < 0.0005), *X* ^2^ (1, *N* = 472) = 17.7, *p* < 0.001.

### MSS Score

The MSS score was compared with the demographics using mean comparison testing (the results are shown in Table 2 ). A Kolmogorov–Smirnov test indicated that the MSS score did not follow a normal distribution, *D* (472) = 0.19, *p* < 0.001. Participants in the age range of 21 to 25 were 1.60 times more likely to score moderate than mild in comparison to the age range of 16 to 20 (95% CI: 1.03–2.49, *p* = 0.035), *X* ^2^ (1, *N* = 325) = 4.5, *p* = 0.035. Participants who scored moderate were 6.81 times more likely to seek medical assistance than those who scored mild (95% CI: 3.69–12.66, *p* < 0.0005), *X* ^2^ (1, *N* = 325) = 43.8, *p* < 0.001. Furthermore, those who scored moderate were 1.66 times more likely to look up symptoms online compared with those who scored mild (95% CI: 1.04–2.64, *p* = 0.031), *X* ^2^ (1, *N* = 325) = 4.7, *p* = 0.031. Participants who scored mild were 1.75 times more likely to think they had an undiscovered illness compared with participants who scored none (95% CI: 1.05–2.93, *p* = 0.032), *X* ^2^ (1, *N* = 294) = 4.6, *p* = 0.032. Additionally, participants who scored mild were 1.75 times more likely to look up their symptoms online compared with those who scored none (95% CI: 1.10–2.79, *p* = 0.017), *X* ^2^ (1, *N* = 294) = 5.7, *p* = 0.017.

**Table 2:** Comparing MSS score with the demographics

		Mean (Max = 21)	Percentage	*p-* Value
Age	17	4.29	20.4	0.008
	18	4.23	20.1	
	19	6.21	29.6	
	20	5.90	28.1	
	21	7.41	35.3	
	22	6.96	33.1	
	23	7.83	37.3	
	24	6.47	30.8	
	25 +	8	38.1	
Gender	Male	5.29	25.2	0.002
	Female	6.95	33.1	
Nationality	UAE national	6.65	31.7	0.675
	Arab	6.47	30.8	
	Non-Arab	5.77	27.5	
Year	Foundation medicine	4.24	20.2	0.001
	Year 1	6.28	29.9	
	Year 2	6.58	31.3	
	Year 3	6.49	30.9	
	Year 4	7.77	37.0	
	Year 5	7.31	34.8	

## Discussion

### Demographics

In this cross-sectional study, it was found that females were 1.86 times more likely to have experienced MSS when compared with males. This finding is consistent with similar studies conducted in Pakistan 6 and Saudi Arabia, 7 which also reported higher levels of MSS in females. This could be explained by the higher proportion of female students in UAE medical colleges. Additionally, it was observed that having greater awareness of MSS could make students more susceptible to developing the syndrome.

### Prevalence of MSS

The results indicated that the prevalence of MSS in the targeted population was 70.8%. The subgroup analysis showed that clinical students were more likely to have MSS than preclinical students. This finding contradicts the result of a study conducted in Saudi Arabia that suggested that MSS is more prevalent among preclinical students. A possible explanation to that could be linked to the massive amounts of knowledge about various diseases medical students are exposed to during their clinical training. 4 As this study revealed, clinical year students believe they are more prone to diseases owing to their field of study. This can contribute to their increased susceptibility of developing MSS due to the psychological stress they are already under. 8

### Knowledge of MSS

A major aim of this study was to investigate the prevalence and explore the knowledge of MSS among medical students, which stemmed mainly from the lack of data in the region. The results revealed that 55.9% of the participants had previous knowledge about MSS. This can be explained by the fact that psychology has become a major subject in the curriculum of medicine, and more students have taken an interest in it. The fact that medical students are also under a huge amount of stress makes them more aware of their psychological well-being and bodily state, 2 which could explain the high awareness about MSS.

### Help-Seeking Behavior

Medical students are more prone to suffer from mental pressure due to various reasons, mainly due to their curriculum and clinical training. The acquisition of extensive medical information throughout their studies compels students to apply their knowledge in their daily life. Additionally, students may start self-treating without obtaining a medical prescription due to their increased sense of confidence in their medical knowledge.

The findings of this study suggested that students who felt their daily activities were being hindered due to the symptoms were more likely to seek medical assistance. However, they may hesitate to seek medical advice if the same student has had a previous interaction with a treating physician that turned out to be the result of MSS. This hesitation can result in the delay of receiving medical care, which might lead to further complications.

### Limitations and Weaknesses

The study followed a cross-sectional design, meaning that the temporal relationship between the exposure and the outcome could not be established as they were assessed at the same time. Additionally, it was only conducted in one medical college in the UAE, which might have limited the variability of the responses. The study population mainly consisted of females, which could have resulted in slight bias in the results. Moreover, conducting the study in languages other than English may provide more insight toward the discussed syndrome. Finally, the survey might have overestimated the prevalence due to recall bias; this could be taken into consideration for further studies to reduce possible bias.

## Conclusion

The results of this study suggest that medical students are more likely to experience MSS as they progress through their years of study. Therefore, programs should be implemented, and psychological support should be extended to medical students to address their needs and promote mental well-being. 9 10 It is also important for more studies to be conducted to further explore the effects of the syndrome on the capabilities and performance of medical students.
